# The Impact of Matrix Metalloproteinase-9 on the Sequential Steps of the Metastatic Process

**DOI:** 10.3390/ijms21124526

**Published:** 2020-06-25

**Authors:** Giovanni Barillari

**Affiliations:** Department of Clinical Sciences and Translational Medicine, University of Rome Tor Vergata, 1 via Montpellier, 00133 Rome, Italy; barillar@uniroma2.it; Tel.: +39-06-7259-6510

**Keywords:** AKT, cancer, EMT, HIV-protease inhibitors, integrins, metastasis, MMP-9, tumor cell invasion

## Abstract

In industrialized countries, cancer is the second leading cause of death after cardiovascular disease. Most cancer patients die because of metastases, which consist of the self-transplantation of malignant cells in anatomical sites other than the one from where the tumor arose. Disseminated cancer cells retain the phenotypic features of the primary tumor, and display very poor differentiation indices and functional regulation. Upon arrival at the target organ, they replace preexisting, normal cells, thereby permanently compromising the patient’s health; the metastasis can, in turn, metastasize. The spread of cancer cells implies the degradation of the extracellular matrix by a variety of enzymes, among which the matrix metalloproteinase (MMP)-9 is particularly effective. This article reviews the available published literature concerning the important role that MMP-9 has in the metastatic process. Additionally, information is provided on therapeutic approaches aimed at counteracting, or even preventing, the development of metastasis via the use of MMP-9 antagonists.

## 1. Introduction

In malignant tumors, transformed cells replicate continuously and infiltrate the tissue from which they originated, replacing preexisting normal cells [1]. Later, proliferating malignant cells eventually diffuse to adjacent and/or distant tissues and organs, giving rise to secondary tumors, i.e., metastases [1]. The latter maintain the phenotypic features of the primary tumor, hence completely upsetting the homeostasis of the human body and rendering the tumor incurable [1].

Cancer metastases occur in sequential steps; in other words, malignant cells: (a) detach from the primary tumor mass; (b) digest the surrounding extracellular matrix (ECM) and migrate through it; (c) penetrate local blood or lymphatic vessels (intravasation), and are transported by the blood or lymph throughout the organism; (d) arrest in the narrow lumen of small vessels, breach the vessel wall, and transmigrate into the extravascular space (extravasation); (e) adapt to the new anatomic site and outgrow [1].

All these steps require cancer cells to break down ECM structures, i.e., the interstitial matrix, which fills the space between the cells of a given tissue, and the basement membranes, sheets of ECM that separate cells lining the epithelium or endothelium from the underlying connective tissue [1,2]. The interstitial matrix is composed of proteoglycans, fibrillar collagens and noncollagenous glycoproteins that are mainly synthesized by stromal cells [2]. Basement membranes are constituted of molecules such as collagen IV, laminin, elastin, fibronectin and tenascin, which are made by epithelial, endothelial or stromal cells [2]. The ECM binds a significant share of cellular products, including growth factors or inflammatory mediators, and stores them in a biologically active form which is preserved from degradation otherwise occurring via serum or tissue protease activity [2]. During cancer cell invasion and metastasis, tumor cells digest the ECM molecules by synthesizing and employing a large variety of enzymes; among them, the matrix metalloproteinases (MMPs) are the main contributors to ECM degradation by tumor cells [3].

MMPs represent a family of proteases which display a common structure consisting of an amino-terminal propeptide domain, a zinc-containing catalytic site, a hemopexin domain granting substrate specificity, and a hinge region which links together the catalytic and hemopexin domains, thereby endowing flexibility upon the enzymatic molecule [4].

The majority of MMPs are released by the producing cells in the extracellular compartment, and other MMPs are bound to the cell membrane. Altogether, these enzymes proteolytically cleave ECM molecules in a redundant fashion [3,4]. Specifically, among released MMPs, stromelysins (MMP-3, MMP-7, MMP-10 and MMP-11) break down most of the ECM components, while interstitial collagenases (MMP-1, MMP-8 and MMP-13) digest collagen I, II and III in close proximity to cells [3,4]. Still regarding released MMPs, the MMP-12 cleaves elastin or fibronectin, and gelatinases (MMP-2 and MMP-9) digest either basement membrane components, such as laminin and collagen IV, or interstitial collagen fragments [3,4]. For their part, the six types of cell surface-bound MMPs, termed membrane-type (MT)-MMPs, can process both released MMPs and ECM molecules [5].

During embryonic life, MMPs are constitutively produced, being important to organ morphogenesis [4,5]; in adult tissues, MMPs are expressed at very low to undetectable levels, being upregulated only during physiological tissue remodeling or in reactive processes, such as inflammation and wound repair [4,5]. In these conditions, the extent and duration of MMP activity is tightly regulated by endogenous inhibitors including serum antiproteases and the Tissue Inhibitors of MMPs (TIMPs) [6]. Specifically, in humans, four types of TIMPs are known, each of which hampers the function of multiple MMPs by binding to their catalytic and/or hemopexin domain [6].

In chronic inflammatory or degenerative diseases, MMPs are overexpressed, altering the TIMP/MMP ratio and leading to a considerable increase in MMP activity [3,7].

Similarly, high MMP levels are found in many types of human cancers, positively correlating with their clinical progression [8,9,10]. Consequently, MMPs are considered as either tumor markers or antitumor therapeutic targets [9,10].

Among MMPs, MMP-9 (also termed gelatinase B) exerts a variety of activities, most of which favor tumor growth and spread [10,11]. However, MMP-9 can also have antitumor effects; this dual behavior depends on the cancer type and/or clinical stage [12,13].

The role that MMP-9 has in the development of the primary tumor has been described elsewhere [14,15,16]. Herein, the available scientific literature concerning the impact that the deregulated expression or activity of MMP-9 might exert on solid tumor metastases is reviewed in the context of a Special Issue of the International Journal of Molecular Sciences, entitled “Tumor Cell Invasion and Metastases”.

## 2. MMP-9 Production or Activity is Regulated at Multiple Levels

**Figure 1:**
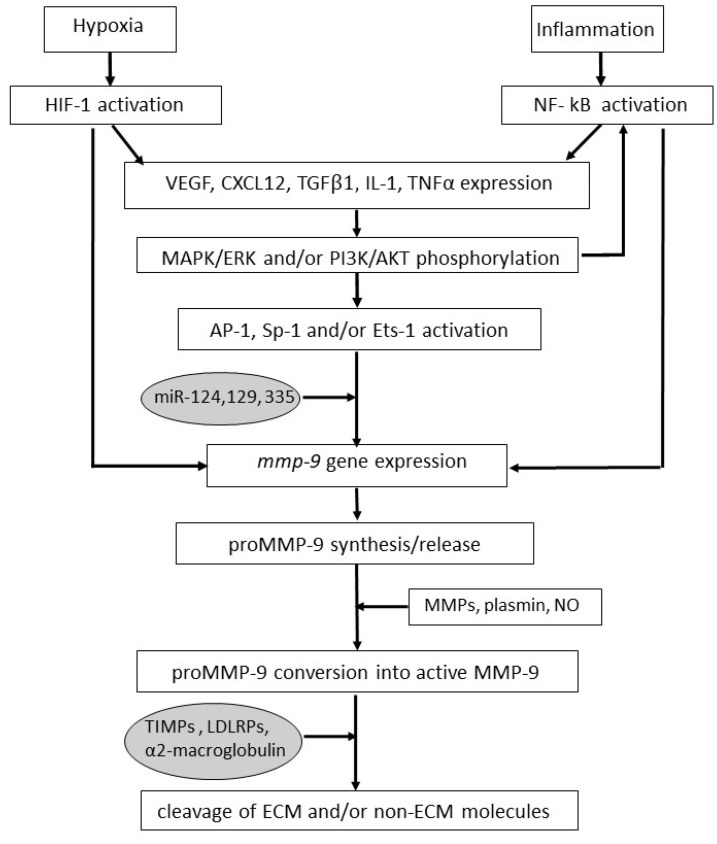
The MMP-9 pathway. In the rectangles, the main molecules and signaling pathways leading to MMP-9 expression or activation are shown; in the ellipses, the endogenous MMP-9 antagonists are listed. Arrows symbolize directions of connections. Abbreviations: AKT, protein kinase B; AP, activator protein; CXCL, CXC chemokine ligand; ECM, extracellular matrix; ERK, extracellular-signal-regulated kinase; HIF, hypoxia-inducible factor; IL, interleukin; LDLRP, low-density lipoprotein receptor-related protein; MAPK, mitogen-activated protein kinase; miR, microRNA; MMP, matrix metalloproteinase; NF-kB, nuclear factor-kappa B; NO, nitric oxide; PI3K, phosphoinositide 3 kinase; Sp, specificity protein; TGF, transforming growth factor; TIMP, tissue inhibitor of matrix metalloproteinase; TNF, tumor necrosis factor; VEGF, vascular endothelial growth factor.

The important role that MMP-9 plays in reactive processes including inflammation and wound repair explains why the enzyme is constitutively expressed only by a few cell types, such as neutrophils and macrophages [4,17]. Other cells (including fibroblasts, osteoblasts and epithelial, endothelial, dendritic and T cells) produce MMP-9 primarily upon stimulation with inflammatory cytokines, chemokines, or growth factors [18,19,20,21,22,23,24]. Among the inducers of MMP-9 expression are interleukin-1, tumor necrosis factor α, epidermal growth factor, transforming growth factor β1, vascular endothelial growth factor (VEGF) and the CXC chemokine ligand 12 (CXCL12) (Figure 1) [18,19,20,21,22,23,24].

Active MMP-9 can directly degrade laminin and, in concert with other enzymes, collagen IV and V, thereby disrupting the basement membrane [4,25,26]. In this context, it should be stressed that similarly to MMP-2, but in contrast with the other members of the family, MMP-9 has a specific collagen-binding domain in its catalytic site [4,25,26]. Through this domain, the enzyme binds and further cleaves the soluble monomers of interstitial collagens which result from ECM degradation started by MMP-1, MMP-8 or MMP-13 [2,3,18]. MMP-9 is also capable of directly digesting elastin, fibronectin or proteoglycans, which contributes to the dissolution of the interstitial matrix [27,28,29]. In addition, MMP-9 cleaves and modifies a variety of non-ECM components, such as chemokines, cytokine receptors, growth factors and intercellular adhesion molecules [30,31,32,33].

The wide spectrum of MMP-9 substrates enables the enzyme to influence several biological processes [4,7,12,13,18,23,30,31,32,33]. For this reason, MMP-9 production and activity are tightly regulated at the transcriptional, posttranscriptional, translational and posttranslational levels, as well as by endogenous serum or tissue inhibitors.

Regarding the MMP-9 gene expression, the promoter region of *mmp-9* gene contains response elements for transcription factors including Activator Protein-1, Specificity protein-1 and Nuclear Factor-kappa B (NF-kB), as well as Ets-1 binding sites; this renders MMP-9 inducible by either inflammatory mediators or growth factors, as mentioned (Figure 1) [18,19,20,21,22,23,24,34,35,36].

In this context, it is noteworthy that transcription factors promoting MMP-9 expression can be repressed by microRNAs, which keeps MMP-9 levels low, unless otherwise required (Figure 1) [37,38].

The MMP-9 protein displays the MMP common core structure, which is constituted by an amino-terminal propeptide, a zinc-binding catalytic site, a linker region and a carboxyl-terminal hemopexin domain [4]. Producing cells secrete MMP-9 as zymogen (proMMP-9), in which the zinc atom present at the catalytic site is bound by a cysteine located in the propeptide domain; this interaction fully inhibits MMP-9 activity, thereby maintaining enzyme latency [4]. Thus, proMMP-9 becomes functionally active only upon the excision of its propeptide; this cleavage is executed by other MMPs (including MMP-1, MMP-2, MMP-3, MMP-7, MMP-10, MMP-13 and MMP-26), or additional enzymes such as plasmin, trypsin, kallicrein, elastases or cathepsins (Figure 1) [4]. Of interest, MMP-9 can also be activated before its release; this occurs at the cell membrane level and is mediated by MT1-MMP and MMP-2 [39].

In the extracellular compartment, MMP-9 protein levels are effectively reduced by low-density lipoprotein receptors-related proteins, which complex MMP-9 and cause its uptake and intracellular degradation by macrophages, fibroblasts, hepatocytes and other cell types (Figure 1) [40,41]. In addition, MMP-9 can be counteracted by endogenous inhibitors including serum α2-macroglobulin or TIMPs (Figure 1) [6,42]. Among the latter, TIMP-1 is particularly effective at inhibiting MMP-9 activity [6]. In this regard, it has to be borne in mind that latent MMP-9 is often released in complex with TIMP-1, with the two proteins being joined at their carboxyl-terminals; when latent MMP-9 is converted into its active form, TIMP-1 is freed, and eventually antagonizes active MMP-9 [6].

Deregulated MMP-9 expression and/or activity causes cellular invasiveness and leads to the growth and clinical progression of a wide variety of human cancers [10,14,15,16,43,44,45,46,47,48,49,50,51,52,53].

In tumor tissues, the control of MMP-9 production is altered or lost due to several causes.

**Figure 2:**
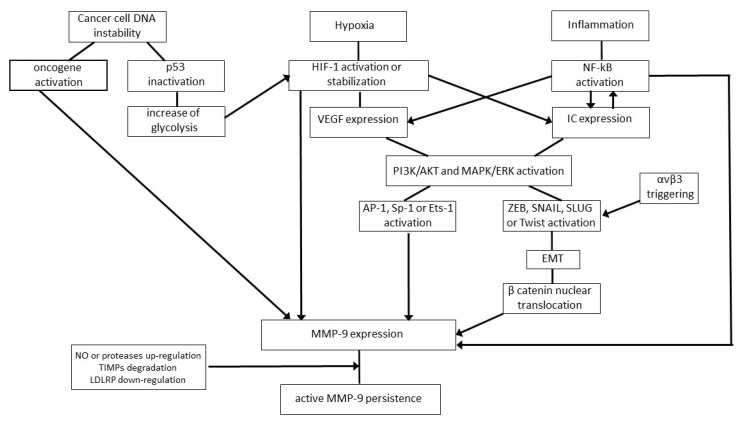
Molecular events promoting the expression and persistence of MMP-9 in tumor tissues. Summary of the molecular pathways leading to MMP-9 overexpression in a wide variety of human tumors. Arrows symbolize directions of connections. Abbreviations: AKT, protein kinase B; AP, activator protein; EMT, epithelial-to-mesenchymal transition; ERK, extracellular-signal-regulated kinase; HIF, hypoxia-inducible factor; IC, inflammatory cytokines; LDLRP, low-density lipoprotein receptor-related protein; MAPK, mitogen-activated protein kinase; MMP, matrix metalloproteinase; NF-kB, nuclear factor-kappa B; NO, nitric oxide; PI3K, phosphoinositide 3 kinase; Sp, specificity protein; TIMP, tissue inhibitor of matrix metalloproteinase; VEGF, vascular endothelial growth factor.

First, cancer cells overexpress MMP-9. This occurs because of both intrinsic and extrinsic mechanisms; the former refers to the unstable genotype of tumor cells featuring the mutation of proto-oncogenes into oncogenes and their abnormal expression, together with the functional impairment of onco-suppressor genes such as p53 (Figure 2) [54,55,56]. In particular, oncogenes directly upregulate MMP-9 expression [54,55], while p53 inactivation triggers MMP-9 synthesis via an increase of glycolysis (Figure 2) [56,57,58].

The extrinsic mechanisms leading to MMP-9 upregulation mainly refer to hypoxia or inflammation, which often coexist at the tumor site (Figure 2) [59,60]. Specifically, when the tumor increases in size, the cells placed in its core area become hypoxic, as they are far from the oxygen-supplying blood vessels [59]. Then, a biologically relevant number of hypoxic tumor cells undergo necrosis, and this sets off an inflammatory response in which leukocytes infiltrate the tumor area and produce cytokines such as tumor necrosis factor α and interleukin-1 or -6 (Figure 2) [60]. Under these conditions, intracellular signaling pathways triggering MMP-9 expression, including the phosphoinositide 3 kinase (PI3K)/protein kinase B (AKT) and the mitogen-activated protein kinases (MAPK)/extracellular-signal-regulated kinases (ERK) pathways, are strongly stimulated, as are the MMP-9 transcription factors Activator Protein-1, Specificity protein-1 or Ets-1 (Figure 2) [34,36,57,61,62]. Moreover, NF-kB is further activated by inflammatory cytokines which are its transcriptional targets, thereby promoting MMP-9 expression by both the transformed and normal cells constituting the tumor mass (Figure 2) [62].

It is worthy of note that in some cancer cell types, the microRNAs hampering MMP-9 expression are downregulated, which additionally increases MMP-9 protein levels and cellular invasion [37]. At the same time, tumor hypoxia triggers the hypoxia-inducible transcription factor (HIF)-1, which, in turn, promotes MMP-9 expression either directly or by activating the expression of growth factors, inflammatory cytokines or chemokines which are able to promote MMP-9 expression (Figure 2) [59]. In addition, HIF-1 triggers the production of nitric oxide, a gaseous mediator capable of activating latent MMP-9 by disrupting the bond between the catalytic zinc and the propeptide domain of the enzyme (Figure 2) [63]. Of interest, MMP-9 and nitric oxide synthases frequently colocalize at the leading edge of invading tumor cells [64].

In addition to being produced by the transformed or normal cells composing the tumor mass, MMP-9 is released in a TIMP-1-free form by neutrophils or macrophages which are recruited to the tumor site during the inflammatory response that accompanies cancer development or progression [4,11,46,60]. Whatever the source of its production, MMP-9 retrieves ECM-bound, sequestered growth factors into a soluble, highly diffusible form [65,66]. The solubilized growth factors, together with inflammatory mediators and additional growth factors released by tumor-infiltrating leukocytes, further stimulate the cells constituting the carcinoma mass to generate additional MMP-9 [11,19,20,21,22,23,24].

Therefore, at the tumor site, MMP-9 produced by cancer cells combines with that synthesized by normal inflammatory, immune, parenchymal or stromal cells; this leads to a dramatic rise of MMP-9 protein levels which is not paralleled by an increase in TIMP synthesis [11,43,44,45,46,47,48,49,50,51,52,53].

Interestingly, MMP-9 clearance by low-density lipoprotein, receptor-related proteins is reduced in tumors compared to normal tissues (Figure 2) [40]. Furthermore, tumor-infiltrating neutrophils produce enzymes, such as trypsin and chymotrypsin, which degrade local, preexistent TIMPs (Figure 2 and Table 1) [67].

The upregulation of MMP-9 expression and/or activity occurring in tumor tissues has a major role in the realization of the sequential steps of the metastatic process.

## 3. MMP-9 Favors Cancer Cell Detachment from the Primary Tumor by Loosening their Adhesive Interactions and Enhancing their Invasive Capabilities

The first step of the metastatic process is the dissolution of the intercellular contacts that maintain the mass of the primary tumor [1].

In this regard, it should be remembered that the adhesiveness among cells composing a given tissue is mediated by adherens junctions and, at a lower efficiency level, by tight and gap junctions [84]. In the epithelial tissue, the main actuator of intercellular adhesions is the epithelial-cadherin, a transmembrane glycoprotein that is the core component of epithelial adherens junctions [84,85,86]. During carcinoma development, growth factors (e.g., epidermal growth factor and transforming growth factor β1) produced by tumor or normal cells trigger the PI3K/AKT and/or the MAPK/ERK pathway; this leads to the activation of the ZEB1, ZEB2, Snail, Slug or Twist transcription factors which, in turn, induce transformed epithelial cells to acquire a mesenchymal phenotype through a process termed epithelial-to-mesenchymal transition (EMT) (Figure 2) [85,86]. The latter can also be promoted by p53 functional loss accompanying tumor onset [56] or by NF-kB and HIF-1, which are activated by inflammation and low oxygen level, respectively [87,88]. EMT endows the transformed cells of the primary tumor with the ability to invade distant tissues [85,86]. In fact, EMT ultimately results in the conversion of nonmotile, polarized epithelial cells into highly mobile, myofibroblast-like cells [85,86]. This phenomenon occurs in a limited way during tissue development or repair (where it is quickly followed by a reversion to the polarized, nonmigratory epithelial phenotype), and it is remarkably exacerbated and prolonged over time during cancer progression and metastasis [85,86].

EMT implicates the downregulation or loss of epithelial cell markers, epithelial-cadherin included, and their replacement with mesenchymal cell markers such as neuronal-cadherin [85,86]. The cadherin switch is followed by a profound reorganization of the cytoskeleton, implying that epithelial keratins are replaced by mesenchymal vimentin [85,86]. Moreover, MMP-2 expression is upregulated, and MMP-9 synthesis is induced [85,86,87,88,89,90]. In fact, the loss of epithelial-cadherin which occurs during EMT causes the nuclear translocation of β-catenin, a cytoplasmic protein which is linked to the actin filaments of the cytoskeleton. Once it is in the nucleus, β-catenin induces NF-kB to activate *mmp-9* gene expression (Figure 2) [89]. Then, MMP-9 completes the cleavage of interstitial collagens, thereby contributing to cancer cell detachment from the primary tumor mass [1,3,4,11].

In essence, during the metastatic process, cancer cells change their state from strongly to weakly adherent [68,85,86].

It has to be pointed out that cells are connected to the ECM through so-called integrins, a family of transmembrane receptors which bind ECM components and links them to the cytoskeleton, thereby modulating not only cellular adhesion, but also cell survival, growth, locomotion and differentiation [68]. Integrins are composed of an α subunit, which binds the ligand, and a β subunit, that transduces the consequent signaling inside the cell via its connection to the cytoskeleton [68]. The majority of integrins can bind several different ligands, and a specific ECM molecule can be recognized by multiple integrins [68]. Usually, an integrin interacts with one ligand rather than another depending on the conformational state of both the integrin and the ligand [68].

Concerning the topic of the present review, it should be emphasized that the rearrangement of the actin cytoskeleton associated with EMT causes integrins to be internalized [68,85,86]. Indeed, MMP-9 could further favor cell detachment because of its ability to shed the integrin β subunit from the cell surface [91]. However, most of the anti-adhesive effect of MMP-9 depends on the fact that this enzyme efficiently degrades the pericellular matrix and basement membrane in cooperation with other MMPs and/or additional proteolytic enzymes [11,18,19,27,28,29].

The breakup of cell–cell or cell–ECM adhesive contacts leads to the invasion of individual cancer cells or multicellular clusters.

Noticeably, many of the matrix-bound growth factors which are released into a soluble form by MMP-9 effectively promote cancer cell locomotion [3,8,11]. Moreover, MMP-9 generates highly chemotactic peptides derived from the degradation of ECM components such as elastin and the collagens [92]. Upon their binding to the integrins, the ECM fragments stimulate not only cellular migration, but also cellular proliferation, thereby strongly contributing to tumor spreading [92].

Tumor cells migrate through cyclical reiterations of frontal elongation and adhesion, followed by cellular constriction and posterior loose [85,86]. Specifically, upon the activation of Rho-GTPases promoted by the chemotactic factors, the cytoskeletal actin polymerizes, resulting in the formation of protrusions at the leading edge of migrating cells [85,86]. Of note, both the integrins and MMPs concentrate at the cellular protrusions; integrins bind to the ECM which supplies a dais upon which cancer cells move, while MMPs break down the ECM barrier, thereby opening a passage for cancer cell dissemination [68,85,86]. Later, the persistent activation of Rho-GTPases triggers cellular constriction and posterior loose, leading to a remarkable frontal elongation of the migrating cancer cells [85,86].

Certainly, tumor cell locomotion depends on somewhat coordinated interaction between integrins and MMPs. 

Overall, MMPs bind to integrins through their hemopexin domain, thereby being anchored on the tumor cell surface [68]. The results from several studies indicate that MMP-9 mainly cooperates with the αvβ3 integrin to promote tumor cell motility [68,69,70]. Specifically, MMP-9 and αvβ3 often colocalize at the invasive front of cancers, where MMP-9 drives cellular invasion by degrading the tumor matrix, thus clearing the way for αvβ3-mediated cancer cell migration (Table 1). In this context, it should be considered that the flexibility of the MMP-9 linker region connecting the catalytic and hemopexin domains optimizes the interaction of migrating cancer cells with MMP-9 substrates [4,68,69,70]. Moreover, the triggering of αvβ3 integrin induces the synthesis of β-catenin [72], which promotes MMP-9 expression [89].

Of interest, the ability of MMP-9 to favor the spread of tumor cells can also be helped by nonintegrin receptors such as CD44 and CD151, which are coexpressed with MMP-9 by highly invasive cancer cells [78,79]. Basically, while CD44 recruits MMP-9 at the protrusions of migrating cells, thereby spatially directing MMP-9 proteolytic activity, CD151 facilitates MMP-9-mediated cancer cell motility by mediating integrin endocytosis at the rear or basal-lateral front of migrating cells (Table 1) [68,78,79]. It is worthy of note that the expression of CD151 and CD44 is upregulated during EMT [79,93].

In conclusion, the mobile/invasive properties of carcinoma cells are strongly enhanced by EMT, which explains the abundance of EMT cells at the invasive front of tumors [85,86]. EMT gives tumor cells a stem cell-like phenotype, endowing them with plasticity, adaptability and a resilience which allow the subsequent steps of metastasis to occur.

## 4. MMP-9 Aids Cancer Cell Trafficking Inside and Outside the Lymphatic or Blood Vessels

Cancer cells can disseminate in the human body either through local invasion or through blood and/or lymph [93].

Specifically, after degrading the basement membrane and the underlying stroma, migrating cancer cells encounter the abluminal side of lymphatic or blood vessels.

Blood capillaries are formed by an endoluminal face constituted by endothelial cells, and an abluminal side composed of a basement membrane and a noncontinuous lining of vascular smooth muscle cells, the so called pericytes [93]. Therefore, to enter the capillaries, tumor cells must degrade the vascular basement membrane via MMP activity, and then pass through endothelial cells [93,94]. In order to intravasate, cancer cells mainly rely on the MMP-9 they produce [93]. Moreover, MMP-9 released by cancer cells which have undergone a full EMT facilitates the intravasation of cancer cells which have been subjected only to a partial EMT [93]. It is worthy of note that while they degrade the blood vessel basement membrane, cancer cells secrete chemokines and/or cytokines which recruit neutrophils to the endothelium [95,96,97]. Activated neutrophils, in turn, release TIMP-1-free MMP-9, which effectively aids cancer cells to breach blood vessel walls [4,11,96,97].

At variance with blood capillaries, the lymphatic ones lack either a basement membrane or pericyte coverage, and display open gaps [93]. Therefore, to enter the lymphatic system, tumor cells do not necessarily have to degrade the vascular wall via MMP proteolytic activity [93].

For both blood and lymphatic vessels, tumor cells transmigrate through the endothelium by diapedesis, a process which implies a high degree of cellular deformability which is accomplished through repeated cycles of cell contraction and expansion [98]. The transendothelial migration of EMT cancer cells is facilitated by both VEGF-promoted vascular permeability and the interaction between MMP-9 and neuronal-cadherin [80,81].

Subsequently, single cancer cells or cancer cell clusters shed into the lymphatic system or blood [98].

Generally, the majority of intravasated tumor cells do not come safely through the hematic or lymphatic stream. This is because circulating tumor cells frequently undergo anoikis, a specific type of cell death resulting from the loss of adhesive interactions with the ECM [99]. This notwithstanding, EMT bestows upon tumor cells the ability to resist anoikis as a part of their highly mobile, poorly adhesive phenotype [99]. Anoikis resistance is mediated by EMT-inducing transcription factors; among them, Twist upregulates the expression of the anti-apoptosis Bcl-2 protein, while Snail activates the AKT survival pathways in carcinoma cells [99].

However, cancer cells overcoming anoikis can be assaulted by immune cells [100]. In this context, MMP-9 protects circulating tumor cells from the lethal actions of natural killer cells or cytotoxic T cells [31,101]. Specifically, on the lymphocyte surface, MMP-9 degrades the receptor for interleukin-2, a growth and function-activating factor for T cells [31]. Similarly, on the tumor cell membrane, MMP-9 proteolitically cleaves natural killer group 2 member D ligands, thereby lowering tumor cell susceptibility to natural killer cells [101]. Moreover, MMP-9 degrades the C1q component of the complement system [102]: this property of MMP-9 could nullify the humoral immunity directed against cancer cells.

While they are transported by blood or lymph, surviving tumor cells can be entrapped within narrow capillaries, especially when tumor cells aggregate in clusters [98].

The mechanisms through which arrested cancer cells come out of the vessels in anatomic sites close to or distant from the primary tumor are very similar to those employed by neutrophils or monocytes to exit from vessels during inflammation [98]. At the beginning, entrapped tumor cells slide toward the margins of the blood or lymph stream; then, they bind with low affinity to selectins, i.e., adhesion receptors expressed on the endothelial cell membrane [103]. This is because carcinoma cells express selectin ligands such as CD24, CD44 or the carcinoembryonic antigen (Table 1) [75,76,78]. Later, the weakly adherent tumor cells tightly attach to endothelial cells via integrin-mediated interactions. For instance, the α4β1 integrin expressed by carcinoma cells can be bound by the vascular cell adhesion molecule-1 which is located on the endothelium (Table 1) [77]. In contrast, the αvβ3 integrin present on endothelial surface binds to L1, a neural cell adhesion molecule which is aberrantly expressed by some cancer cell types (Table 1) [72].

On the surface of carcinoma cells which have adhered to the endothelium, the CD44 receptor anchors activated MMP-9 and redistributes it at the invading edge of tumor cells, together with the highly proteolytic MT1-MMP [78]. This potentiates and localizes MMP-9-promoted breakdown of the vessel wall required for tumor cell extravasation (Table 1) [104].

## 5. MMP-9 Favors Tumor Cell Adaptation to the Microenvironment of the Secondary Site, Hence Effectively Participating in the Establishment of Metastasis

When disseminated tumor cells arrive at distant organs, they find a microenvironment which differs from that of the organ where the primary tumor developed. Consequently, many of the cancer cells which have reached the secondary site undergo apoptosis, while others enter a state of dormancy in which they stay alive but do not proliferate [2].

Indeed, the successful establishment of the metastasis depends on the formation of a permissive environment within the target secondary organ. Such an environment, termed the premetastatic niche, can be built up by primary tumor cells via the release of molecules supporting the survival and growth of metastatic tumor cells [2]. These molecules diffuse throughout the organism and reach distant organs either free or encapsulated in the exosomes shed from the primary tumor cell surface [2].

Exosomes are small extracellular vesicles of endosomal origin which are released by either cancer cells or normal cells, and contain cellular molecules including nucleic acids, protein or lipids [2].

It is well established that upon their release by a given cell, exosomes interact with another neighbor or distant cell through cellular uptake, fusion with the cell membrane or binding to cell surface receptors [2]. In the context of the metastatic process, exosomes cause cancer and normal cells to communicate with each other in order to build up the premetastatic niche [2].

In addition to exosomes, primary tumor cells release nonencapsulated growth factors (e.g., VEGF or transforming growth factor β1), cytokines (e.g., tumor necrosis factor α) or chemokines (e.g., CXCL12) which recruit to the target metastatic site(s)’ highly mobile, poorly differentiated cells which are normally involved in tissue repair, such as mesenchymal stem cells and bone marrow-derived myeloid cell precursors [105].

The exosomes, growth factors, cytokines or chemokines released by primary tumor cells, myeloid cell precursors and mesenchymal stem cells modify the phenotype of fibroblasts and macrophages which reside in the metastasis target organ(s). Subsequently, activated fibroblasts subvert the composition, structure and stiffness of the secondary site ECM by secreting collagens, fibronectin, tenascin or periostin. These newly produced ECM molecules activate integrin-mediated signaling pathways in the incoming cancer cells, leading to cancer cell survival and proliferation [106]. For their part, activated macrophages release MMP-9 which further remodels the structure of local ECM to favor the homing of disseminated cancer cells at distant sites [107,108,109]. Later, exosomes released by normal cells residing in metastasis target organ(s) upregulate MMP-9 expression in the incoming cancer cells [110,111,112,113]. Then, exosomes released by tumor cells augment MMP-9 production by partner tumor cells or normal cells which reside in the colonized site(s) [114,115,116,117,118,119,120,121]. This is because exosomes contain microRNAs, chemokines, chemokine receptors, ECM molecules or adhesion receptors which activate transcription factors (e.g., NF-kB) and/or signaling pathways (e.g., MAPK/ERK and PI3K/AKT), leading to MMP-9 expression in target cells [110,111,114,116,117,118,119,120,122,123,124]. Moreover, exosomes can store active MMPs [124].

The increase in MMP-9 levels at secondary sites helps metastasis establishment and outgrowth by both mediating cancer cell infiltration of the foreign tissue and releasing protumorigenic factors from the ECM [65,66,92,116].

Soon after they reach the new anatomic site, many of the cancer cells undergo mesenchymal-to-epithelial transition (MET); this implies that carcinoma cells reexpress epithelial adherens junctions and lose their EMT, migratory phenotype [93].

MET is promoted by microRNAs (e.g., the miR-200) repressing the function of EMT-inducing transcription factors [93]. In addition, variations in local ECM composition, such as that following proteoglycan depositing by myeloid cell precursors, are particularly effective at promoting MET [125]. Moreover, normal cells residing in secondary sites release integrin-containing exosomes which fuse with the incoming neoplastic cells, thereby rendering them capable of adhering to the foreign tissue ECM, and subsequently surviving and proliferating [126].

In essence, returning to the epithelial phenotype favors metastatic cell proliferation, which is necessary for the development of a clinically relevant metastasis [127,128,129]. Thus, under the influence of microenvironmental stimuli, cancer cells switch from EMT to MET, with both phenotypes being necessary for the establishment of metastasis.

However, for a metastasis to settle successfully, local antitumor immune responses must be neutralized. Noticeably, MMP-9 counters the anticancer activity of tumor-infiltrating cytotoxic T lymphocytes or natural killer cells [130,131,132].

The survival and growth of disseminated cancer cells lead to the development of micrometastases in which the number of proliferating tumor cells is close to that of tumor cells undergoing death [133]. The share of proliferating cancer cells increases, and metastases grow in size when new blood vessels supplying oxygen and nutrients to metastatic cancer cells are formed [133]. This is accomplished through a multistep process termed angiogenesis [73,74], whereby endothelial cells lining the endoluminal face of a preexisting local vessel first degrade the vessel basement membrane, and then migrate to the perivascular matrix while proliferating [73,74]. This gives rise to endothelial cell solid cords that will ultimately differentiate into hollow tubes, allowing blood flow [73,74]. These sequential events are orchestrated by a variety of growth factors, ECM-degrading enzymes and adhesion receptors [73,74]. Among these molecules, MMP-9 plays a prominent role in angiogenesis by cooperating with VEGF and the αvβ3 integrin (Table 1) [11,73,74,134,135,136]. Specifically, the MMP-9 produced by metastatic tumor cells and metastasis-infiltrating neutrophils and/or macrophages breaks down the vessel basement membrane and perivascular matrix, thereby generating ECM fragments [11,20,26,27,28,29,92,134]. The latter, in turn, trigger αvβ3-mediated endothelial cell migration (Table 1) [135]. At the same time, MMP-9 retrieves ECM-bound VEGF in a soluble form which mediates endothelial cell proliferation and endothelial cords differentiation, occurring respectively in the initial and final stages of angiogenesis (Table 1) [65,73,74,136].

Finally, MMP-9 mobilizes endothelial cell precursors from the bone marrow so that they can incorporate into the nascent vasculature of metastasis and then differentiate into mature endothelial cells (Table 1) [82].

It should be remembered that MMP-9 is also important for the angiogenic switch that causes either the growth or the metastatic dissemination of the primary tumor [73,74]. Moreover, in tumor tissue, MMP-9 triggers the formation of new lymphatic vessels, providing additional routes for cancer metastasis [137].

## 6. MMP-9 Inhibitors

Upon their establishment and outgrowth, cancer metastases hamper the function of the colonized organs, which compounds the harmful effect of the primary tumor and significantly worsens the patient’s health [1]. Under these circumstances, surgical procedures or combined chemo(radio)therapy are not effective, even though they can successfully treat the majority of nonmetastatic primary tumors [1].

In view of the role that MMPs play in cancer dissemination, during the past thirty years, synthetic MMP inhibitors have been developed and tested for therapeutic efficacy (Table 2).

Peptides chelating MMP catalytic zinc via a molecular mimicry of collagens (e.g., BATISTAMAT or MARIMASTAT) have been synthesized and found to counteract the metastatic process in animal models; their use has also been studied in cancer patients [138]. The results from phase II or III clinical trials were not positive because of the poor bioavailability or pharmacokinetics and the dose-limiting musculoskeletal side effects of these drugs (Table 2) [138]. In this regard, it must be stressed that first-generation MMP inhibitors impair the function of all the members of the MMP family at the same time, due to homology between the catalytic domains of these enzymes [4,138]. Indeed, the use of multiple drugs simultaneously counteracting all MMPs is not recommended. In fact, these drugs hamper the physiologic processes (e.g., wound healing) that MMPs mediate in a redundant fashion [3,4].

Based on these clinical findings, structural analyses have been performed in order to design compounds specifically inhibiting one or a few MMPs; the results indicated that near MMP active site, an exosite is present which is variable among the MMPs and confers specificity for substrate binding [139]. Hence, additional MMP inhibitors (e.g., TANOMASTAT) have been developed to bind to that exosite, and then chelate the catalytic zinc in a small number of MMPs, MMP-9 included [139]. Unfortunately, these MMP inhibitors have been ineffective in cancer patients (Table 2) [139].

Further efforts have been specifically directed against MMP-9, considering the impact that this enzyme has on cancer metastasis. Results from clinical investigations indicate that natural compounds inhibiting MMP-9 expression or activity, such as QUERCETIN and CURCUMIN, enhance the efficacy of standard antitumor chemotherapy and reduce its side effects (Table 2) [140,151]. Concerning different anti-MMP-9 strategies, humanized anti-MMP-9 monoclonal antibodies (ANDECALIXIMAB) have been explored in gastric cancer patients, and found to be safe (with no indication of musculoskeletal side effects) and moderately effective when combined with classical cytotoxic drugs (Table 2) [141]. In contrast, a cyclic peptide (CILINGITIDE) selectively antagonizing αv integrins did not inhibit glioblastoma progression [152], despite αvβ3 actively cooperating with MMP-9 to promote tumor cell dissemination [68,69,70,71]. Similarly, the antibiotic DOXYCYCLINE has not been of significant benefit in patients affected by metastatic breast carcinoma (Table 2) [142]—even though the drug is effective against breast carcinoma in animal models [153,154]—or it increases TIMP-1 levels in treated patients [155].

The poor performance of DOXYCYCLINE in anticancer clinical trials is particularly disappointing, as reusing extant medicines is a rapid approach to elaborating antitumor pharmaceuticals [156].

In this regard, however, results from clinical-epidemiological studies have indicated that drugs which have been employed for a long time in the therapy of human immunodeficiency virus (HIV)-1 infection exert direct antitumor activities that are independent of the drug antiviral or immune-reconstituting effects [48,157].

In particular, compounds functionally impairing the HIV aspartyl protease or reverse transcriptase, two enzymes which are pivotal in the replication or infectivity of the virus, have significantly reduced the incidence or progression of HIV-associated tumors [48,157,158]. Further clinical work has indicated that HIV protease or reverse transcriptase inhibitors might also hamper tumor progression in HIV-negative patients (Table 2) [143,159,160,161].

In this context, preclinical studies have shown that HIV protease inhibitors effectively reduce the growth of highly prevalent human tumors in animal models in the absence of HIV infection [162,163]. In fact, these anti-HIV drugs inhibit events which are key to cancer growth and dissemination, such as ECM breakdown and cell invasion, glycolysis and cell locomotion, fatty acids oxidation and cell proliferation, thereby converting invasive cancer cells into nonmotile cells [164,165,166,167,168].

It is worthy of note that HIV protease inhibitors such as ritonavir, saquinavir, indinavir and lopinavir strongly reduce MMP-9 expression and/or activity in normal or cancer cells and in treated patients [20,169,170,171,172,173,174]. This is because HIV protease inhibitors repress the MAPK/ERK and/or PI3K/AKT signaling pathways, thereby functionally impairing transcription factors targeting the *mmp-9* gene, including NF-kB, Specificity protein-1 or the Fra-1 element of the Activator Protein-1 transcriptional complex [164,169,175,176,177,178,179,180,181,182,183,184,185,186,187,188,189,190,191,192,193,194,195,196]. It should be noted that the AKT activation process starts when PI3K promotes the production of phosphatidylinositol-3,4,5-trisphosphate (PIP3); this is followed by AKT recruitment to the cell membrane and AKT phosphorylation on Thr308 and Ser473 by the phosphoinosotide-dependent kinase-1 and the mammalian target of rapamycin, respectively [191]. In this context, the HIV-protease inhibitor nelfinavir impairs AKT phosphorylation by hampering AKT recruitment to the cell membrane. The finding that nelfinavir does not reduce PIP3 production suggests that the drug may lower PIP3 sensing by AKT [190,191]. Moreover, nelfinavir can impair the mammalian target of rapamycin, which is both an activator and a downstream target of AKT [182]. For its part, the HIV-protease inhibitor ritonavir dephosphorylates AKT by reducing the levels of heat shock protein 90 [183,186]. Finally, with regard to MAPK activation, it should be emphasized that the HIV-protease inhibitors indinavir and nelfinavir dephosphorylate mitogen-activated protein kinase 1/2, which prevents the downstream activation of MAPKs [178,180]. Specifically concerning nelfinavir, it dephosphorylates mitogen-activated protein kinase 1/2 by augmenting protein-phosphatase-2 activity [180].

Additional studies suggest that HIV protease inhibitors could impair MMP-9 activity also because of their ability to upregulate TIMPs levels [171,197], diminish the production of nitric oxide and reduce the expression of MT1-MMP [192,198,199,200,201]. The fact that they reduce the expression of MMPs while increasing that of TIMPs makes HIV-protease inhibitors similar to natural compounds that perform these actions thanks to the same ability to hamper the AKT and MAPK signaling pathways [202,203]. Also, synthetic antagonists of α1-antitrypsin, an endogenous protease inhibitor which is overexpressed in tumor tissues, downregulate MMPs and, at the same time, upregulate TIMPs via PI3K/AKT inhibition [204]. It is worthy of note that the HIV-protease inhibitor ritonavir reduces α1-antitrypsin protein levels in treated patients [205]. Future work will evaluate whether α1-antitrypsin is involved in the antitumor activities of HIV-protease inhibitors.

Consistent with their antagonist effect on MMPs, HIV protease inhibitors block ECM invasion by cancer cells or endothelial cells, thereby halting both tumor dissemination and angiogenesis [20,48,157,162,163,169,192,206].

The anti-angiogenic activity of HIV-protease inhibitors is particularly interesting, since combining standard chemo(radio)therapy with anti-angiogenic strategies has been shown to improve the prognosis of cancer patients, consistent with the fact that angiogenesis sustains tumor cell growth and spread throughout the organism [74,207].

In this context, it has to be highlighted that due to their repressive activity on HIF-1 or NF-kB, HIV-protease inhibitors downregulate the expression of VEGF, the most potent inducer of tumor angiogenesis [175,176,177,193].

Indeed, VEGF antagonists including multikinase inhibitors and antibodies targeting VEGF or its type-2 receptor represent a group of anti-angiogenetic drugs that is widely used at present [73]. However, the extended application of these VEGF inhibitors causes adverse effects encompassing high blood pressure, impaired wound healing or hemostasis, proteinuria and gastrointestinal ulceration [73]. Moreover, long-term-treated patients become insensitive to these drugs, as the inhibition of the VEGF pathway triggers the production of other angiogenic factors [208]. In this respect, HIV-protease inhibitors promise to be more effective than classical VEGF antagonists, as they halt angiogenesis by blocking MMP proinvasive activity, regardless of the involved angiogenic factor [162,192,206].

Although it largely contributes to the angiogenic switch required for metastasis outgrowth, MMP-9 can generate anti-angiogenic compounds (e.g., tumstatin and endostatin) resulting from the proteolytic cleavage of ECM molecules [12,13]. In this regard, however, one should consider that inhibiting MMP-9 is unlikely to significantly reduce the levels of anti-angiogenic ECM fragments, since they can also be generated by other MMPs [12,13].

Clinical trials recruiting HIV-infected or uninfected individuals have shown that when HIV-protease inhibitors are administered together with standard chemo(radio)therapy, they promote the regression of advanced carcinomas [209,210]. In contrast, HIV-protease inhibitor monotherapy is active against early- but not late-stage tumors [48,143,159,211,212,213,214]. However, it should be noted that despite their multiple antitumor effects, first generation HIV-protease inhibitors (e.g., ritonavir, nelfinavir and lopinavir) augment glycemia and/or lipemia in treated patients (Table 2) [144,145]. Concerning second generation HIV-protease inhibitors (e.g., darunavir and atazanavir), they display only minor toxicities [215], but their antitumor activities are not yet well-defined.

The HIV-reverse transcriptase inhibitors zidovudine and lamivudine can downregulate MMP-9 expression in normal or tumor cells, weaken MAPK/ERK or PI3K/AKT phosphorylation, reduce nitric oxide production and impair angiogenesis [174,216,217,218]. In contrast, other HIV-reverse transcriptase inhibitors (e.g., tenofovir and efavirenz) are able to exert either pro- or anti- tumor activities [217,219].

Results from medical tests indicate that, when combined with standard antitumor cytotoxic drugs, zidovudine is effective against T cell leukemia (Table 2) [146], while efavirenz slows prostate carcinoma progression and lamivudine reduces the incidence of hepatocellular carcinoma [160,161].

Nausea, vomiting, diarrhea, headache and/or rash often occur in HIV-positive individuals taking HIV-reverse transcriptase inhibitors (Table 2) [147,220,221]. Moreover, the long-term use of these drugs has been reported to cause neutropenia, anemia, myopathy, and liver or kidney malfunction (Table 2) [147,220,221].

Therefore, current work is exploring the antitumor activity of AMD3100, an antagonist of the CXC chemokine receptor 4 (CXCR4) which has been in use for some time for HIV-infected individuals, as it efficiently counteracts virus entry into target cells [222]. Reassuringly, AMD3100 clinical adoption entails only mild side effects, including transient musculoskeletal pain and/or gastrointestinal disturbances (Table 2) [150].

The rational for the use of AMD3100 in oncology is that CXCR4 binding by natural ligands, such as the CXCL12 chemokine, triggers PI3K/AKT, thereby promoting MMP-9 expression [223] and cancer cell invasion/dissemination [224,225,226,227,228]. Consistently, AMD3100 has reduced MMP-9 production and hampered cancer metastasis in preclinical models [229].

At present, AMD3100 is successfully being used in the treatment of hematological malignancies (Table 2) [148,149]. Indeed, results from observational studies encourage the use of AMD3100 against a broader spectrum of neoplasms, solid tumors included [230,231,232].

## 7. Conclusions and Future Directions

The expression and/or activity of MMP-9 are increased in different types of human tumors, playing a major role in their progression [43,44,45,46,47,48,49,50,51,52,53]. In particular, MMP-9 is pivotal in all steps of the metastatic process, in that it strongly contributes to the ability that cancer cells have to: (1) disrupt the cell-to-cell or cell-to-ECM interactions that maintain the primary tumor mass; (2) penetrate local ECM and vessels; (3) exit the vessel and migrate into the extravascular space; and (4) survive and proliferate in foreign tissue [1,3,10,11].

Accordingly, MMP-9 expression level is believed to reliably monitor cancer clinical progression [44,45,46,47,48,49,50,51,52,53].

Moreover, MMP-9 is considered a valid antitumor target. In this respect, a variety of approaches have been pursued, although much work remains to be done. In particular, broad-spectrum MMP inhibitors have failed in clinical trials due to their lack of selectivity and dose-limiting, severe side effects [138]. For their part, compounds directed at reducing MMP-9 activity have not reached the required efficacy [139,140,141,142,151].

The good news is that inhibitors of HIV protease or reverse transcriptase and CXCR4 antagonists, which have been used for many years to counter HIV infection, effectively inhibit MMP-9 expression and tumor cell dissemination [20,157,158,159,160,161,169,170,171,172,173,174,216,227].

In this regard, results from preclinical studies indicate that HIV-protease inhibitors compromise a wide variety of metabolic or signaling pathways which are pivotal in cancer cell survival, proliferation and locomotion [48,157]. In contrast, the anticancer activities of HIV-reverse transcriptase inhibitors appear to be more limited, as these drugs impair some protumor pathways while favoring others [174,216,217,218,219].

Probably one of the most relevant anticancer activities of HIV-protease inhibitors is their ability to hamper the AKT signaling pathway [183,184,185,186,187,188,189,190,191]. In this respect, HIV-protease inhibitors may resemble novel AKT inhibitors such as IDELALISIB and COPANLISIB, which were recently approved for clinical use in various types of cancer [233]. However, one should consider that, at variance with novel AKT inhibitors, HIV-protease inhibitors have been utilized for a long time in the treatment of HIV-infected individuals, and their tissue distribution and pharmacokinetics are well known [215].

As for HIV-protease inhibitors, antagonists of the CXCL12/CXCR4 axis also switch off the AKT-MMP pathway; however, HIV-protease inhibitors reduce the levels of both total and phosphorylated AKT [175,176,183,184,185,186,187,188,189,190,191], while CXCR4 antagonists cannot prevent CXCL12 binding to other chemokine receptors which are able to turn on AKT phosphorylation and MMP expression [83].

Results from clinical trials indicate that HIV-protease inhibitors are more efficacious in the early (premetastatic) stages of tumor progression [48,143,157,159]; this observation suggests that the HIV-protease inhibitors could be explored for metastases prevention in early-stage cancers overexpressing MMP-9. Although their repurposing for tumor therapy appears feasible, the long-term administration of HIV-protease inhibitors causes harmful side effects, such as hyperlipemia and hyperglycemia [144,145,215]. Based on structural analyses, preclinical studies and clinical tests, further research should conceive and evaluate HIV-protease inhibitor analogs which are endowed with activities that are as restricted as possible to the anticancer one.

It is well established that the antitumor efficacy of HIV-protease inhibitors augments when they are administered in combination with standard chemotherapy or radiotherapy [210,234,235]. Given the role that inflammation and the immune response play in promoting and contrasting tumor growth, respectively, in the near future, clinical trials should evaluate whether the anticancer activities of HIV-protease inhibitors could be potentiated also by anti-inflammatory drugs and/or compounds enforcing antitumor immune surveillance. Likely candidates could be cyclooxygenase-2 inhibitors or antibodies directed against negative regulators of T cell responses, as both of these classes of drugs have been shown to be of significant benefit in cancer patients [236,237].

## Figures and Tables

**Table 1:** Molecules enhancing MMP-9 activity in tumor tissues.

**M** **olecule**	**A** **ction**
Nitric oxide	Contributes to proMMP-9 conversion into active MMP-9 [63,64]
Tryspin or chymotrypsin	Degrade TIMPs [67]
MMP-2 and MT1-MMP	Activate cell surface-bound MMP-9 [39]
Plasmin or MMP-1, 3, 7, 10, 13 and 26	Activate released MMP-9 [4]
αvβ3	Drives tumor or endothelial cell migration toward chemotactic factors generated and/or released by MMP-9 [68,69,70,71]; Facilitates MMP-9-promoted cancer cell extravasion [72]; Cooperates with MMP-9 to induce new vessel formation [73,74]
α4β1, CEA or CD24	Facilitate MMP-9-promoted cancer cell extravasion [75,76,77].
CD44	Facilitates MMP-9-promoted cancer cell extravasion and spatially directs MMP-9 proteolytic activity [78].
CD151	Facilitates MMP-9-promoted cancer cell locomotion [79].
Neuronal-cadherin	Facilitates the dissemination of cancer cells promoted by MMP-9 [80]
VEGF	Cooperates with MMP-9 to induce cancer cell transendothelial migration [81], and new vessel formation [73,74,82]
CXCL12	Cooperates with MMP-9 in promoting new vessel formation [73,74,83]

List of enzymes (trypsin, chymotrypsin, plasmin, MMPs or MT-MMP), cell surface receptors (αvβ3, α4β1, CEA, CD24, CD44, CD151 or neuronal-cadherin), growth factor (VEGF), chemokine (CXCL12) and the gaseous mediator (nitric oxide) expressed in cancer tissues, where they enhance the protumor activities of MMP-9. Abbreviations: CD, cluster of differentiation; CEA, carcinoembryonic antigen; CXCL, CXC chemokine ligand; MMP, matrix metalloproteinase; MT-MMP, membrane type-matrix metalloproteinase; TIMP, tissue inhibitor of matrix metalloproteinase; VEGF, vascular endothelial growth factor. References with specific information are shown in square brackets.

**Table 2:** Mechanism of action, clinical outcomes and toxicities of compounds inhibiting MMP-9 expression and/or activity.

Drug	Class	Mechanism of Action	Clinical Outcomes	Toxicities
MARIMASTAT	Broad-spectrum MMPI	Chelates MMP catalytic zinc	Does not improve OS of glioblastoma, NSCLC and pancreatic or colorectal ca pts [138]	Musculoskeletal pain [138]
TANOMASTAT	Narrow-spectrum MMPI	Binds MMP exosite	Does not improve OS of NSCLC, pancreatic or ovarian ca pts [139]	Nausea, vomiting [139]
CURCUMIN	Plant extract	Reduces MMP-9 expression	Improves the efficacy of chemotherapy in prostate ca pts [140]	None [140]
ANDECALIXIMAB	Humanized mAB	Neutralizes MMP-9	Improves OS and reduces tumor size in gastric ca pts [141]	Nausea, vomiting, fatigue [141]
DOXYCYCLINE	Antibiotic	Reduces MMP-9 expression	Does not improve OS of breast ca pts [142]	Nausea, vomiting, diarrhea [142]
LPV-RTV	HIV-PI	Inhibits the AKT-MMP-9 pathway	effective against CIN [143]	Increase in glycaemia and/or lipemia [144,145]
AZT	HIV-RTI	Reduces MMP-9 expression	Improves the efficacy of chemotherapy in T cell leukemia pts [146]	Headache, nausea, vomiting, neutropenia, anemia, hepatotoxicity, myopathy [147]
AMD3100	CXCR4 antagonist	Inhibits the CXCL12-AKT-MMP-9 pathway	Effective against hematological malignancies [148,149]	Musculoskeletal pain, gastrointestinal disturbances [150]

Here, we summarize the biological and clinical effects of MMP-9 antagonists approved for therapeutic use in humans. The table shows the activities of a drug selected (because of its ability to effectively antagonizr MMP-9) for each of the following chemical-pharmacological classes: broad or narrow-spectrum synthetic MMP inhibitors, plant extracts, anti-MMP-9 antibodies, antibiotics, chemokine receptor antagonists, and inhibitors of the HIV-protease or reverse transcriptase. Abbreviations: AKT, protein kinase B; AZT, zidovudine; ca, carcinoma; CIN, cervical intraepithelial neoplasia; CXCL, CXC chemokine ligand; CXCR, CXC chemokine receptor; HIV, human immunodeficiency virus; LPV, lopinavir; mAB, monoclonal antibody; MMP, matrix metalloproteinase; MMPI, matrix metalloproteinase inhibitor; NSCLC, nonsmall cell lung carcinoma; OS, overall survival; PI, protease inhibitor; pts, patients; RTI, reverse transcriptase inhibitor; RTV, ritonavir. References with specific information are given in square brackets.

## Abbreviations

AKT	protein kinase B
CXCL	CXC chemokine ligand
CXCR	CXC chemokine receptor
ECM	extracellular matrix
EMT	epithelial-to-mesenchymal transition
ERK	extracellular signal-regulated kinase
HIF	hypoxia inducible factor
HIV	human immunodeficiency virus
MAPK	mitogen activated protein kinase
MET	mesenchymal-to-epithelial transition
MMP	matrix metalloproteinase
MT-MMP	membrane type-matrix metalloproteinase
NF-kB	nuclear factor-kappa B
PI3K	phosphoinositide 3 kinase
PIP3	phosphatidylinositol-3,4,5-trisphosphate
TIMP	tissue inhibitor of matrix metalloproteinase
VEGF	vascular endothelial growth factor
